# UAV and Deep Learning for Automated Detection and Visualization of Façade Defects in Existing Residential Buildings

**DOI:** 10.3390/s25237118

**Published:** 2025-11-21

**Authors:** Yue Fan, Jinghua Mai, Fei Xue, Stephen Siu Yu Lau, San Jiang, Yiqi Tao, Xiaoxing Zhang, Wing Chi Tsang

**Affiliations:** 1 State Key Laboratory of Subtropical Building and Urban Science, Center for Human-Oriented Environment and Sustainable Design, School of Architecture and Urban Planning, Shenzhen University, Shenzhen 518060, China; yfan@szu.edu.cn (Y.F.); 2310325038@email.szu.edu.cn (J.M.); 2 Faculty of Architecture, The University of Hong Kong, Hong Kong 999077, China; ssylau@hku.hk; 3 Guangdong Key Laboratory of Urban Informatics, Shenzhen University, Shenzhen 518060, China; jiangsan@szu.edu.cn; 4 Shenzhen Wuce Geo-Information Technology Co., Ltd., Shenzhen 518000, China; zhangxiaoxing@wucesi.com; 5 School of Science and Technology, Hong Kong Metropolitan University, Hong Kong SAR, China; awctsang@hkmu.edu.hk

**Keywords:** unmanned aerial vehicle (UAV), deep learning, coordinate transformation, building façade defect segmentation, mapping, high-density cities

## Abstract

As urbanization accelerates, façade defects in existing residential buildings have become increasingly prominent, posing serious threats to structural safety and residents’ quality of life. In the high-density built environment of Shenzhen, traditional manual inspection methods exhibit low efficiency and high susceptibility to omission errors. This study proposes an integrated framework for façade defect detection that combines unmanned aerial vehicle (UAV)-based visible-light and thermal infrared imaging with deep learning algorithms and parametric three-dimensional (3D) visualization. Three representative residential communities constructed between 1988 and 2010 in Shenzhen were selected as case studies. The main findings are as follows: (1) the fusion of visible and thermal infrared images enables the synergistic identification of cracks and moisture intrusion defects; (2) shooting distance significantly affects mapping efficiency and accuracy—for low-rise buildings, 5–10 m close-range imaging ensures high mapping precision, whereas for high-rise structures, medium-range imaging at approximately 20–25 m achieves the optimal balance between detection efficiency, accuracy, and dual-defect recognition capability; (3) the developed Grasshopper-integrated mapping tool enables real-time 3D visualization and parametric analysis of defect information. The Knet-based model achieves an mIoU of 87.86% for crack detection and 79.05% for leakage detection. This UAV-based automated inspection framework is particularly suitable for densely populated urban districts and large-scale residential areas, providing an efficient technical solution for city-wide building safety management. This framework provides a solid foundation for the development of automated building maintenance systems and facilitates their integration into future smart city infrastructures.

## 1. Introduction

### 1.1. Background

Urban renewal of existing residential areas has become a common challenge faced by many countries worldwide. With the acceleration of urbanization, problems such as building aging and safety hazards in high-density neighborhoods have become increasingly prominent. These issues not only affect the quality of life for residents but also directly relate to public safety and sustainable urban development. To address this, many countries have begun to introduce advanced technologies—such as unmanned aerial vehicles (UAVs), deep learning, and Building Information Modelling (BIM)—into the renovation of existing buildings to enhance diagnostic accuracy, evaluation, and maintenance efficiency.

As a frontier city of China’s reform and opening-up, Shenzhen has long attracted a large inflow of population and resources, making it a typical high-density city. The scarcity of urban land resources has led to the prevalence of high-rise buildings and compact residential patterns. In older residential areas in particular, historical and planning constraints have resulted in high building density, large floor area ratios, and narrow spacing between buildings. These neighborhoods are characterized by concentrated living environments, deteriorating building quality, and excessive energy consumption, often accompanied by low design standards, outdated material technologies, and insufficient maintenance. Building safety, functionality, and sustainability have therefore become critical concerns. Statistics show that in Shenzhen, old residential communities with a floor area ratio greater than 2.5 cover about 105 square kilometers, accounting for 21.9% of the city’s residential land. More than 36% of houses were built before the year 2000, and 1622 residential communities have been listed for renovation.

Traditional diagnostic and evaluation workflows face multiple challenges when applied to high-density urban neighborhoods. These include limited accessibility for inspectors, low efficiency, the risk of missing defects, safety hazards in high-altitude operations, as well as high costs associated with equipment deployment and maintenance. In addition, limitations in data analysis and processing hinder effective adaptation to the complex and dynamic conditions of dense urban environments. More critically, the lack of data-driven and intelligent methods to transform experiential assessments into quantitative evaluation and prediction restricts the objectivity and efficiency of renovation diagnostics. This calls for the exploration of new technologies and methodologies.

In response to these challenges, this study proposes an intelligent diagnostic method that integrates UAV aerial imagery with deep learning for detecting façade defects in aging residential buildings within high-density areas of Shenzhen. A DJI M3T UAV was employed for oblique photography, with carefully planned flight routes to collect both visible-light and thermal infrared imagery. At the algorithmic level, a deep learning-based model was developed to identify two common types of façade defects: cracks and water leakage. Crack detection was performed using visible-light images, while leakage was identified through thermal infrared imaging of abnormal temperature patterns. These two defects are representative and frequently observed under the humid climatic conditions of southern China.

Furthermore, a three-dimensional spatial mapping method was established to project the two-dimensional detection results onto a 3D building model. By employing coordinate transformation, distortion correction, and ray-tracing techniques, the system generates an intuitive visualization of defect distribution. Case studies conducted in Huaqiaocheng East, Shennan Garden, and Huifang Garden in Shenzhen validated the effectiveness and practicality of the proposed method in complex high-density urban environments. This research provides a novel technological pathway for intelligent building diagnostics and informed decision-making in urban renewal.

### 1.2. Literature Review

In recent years, many scholars have advanced building inspection methods by integrating drone imagery, deep learning algorithms, and non-destructive testing (NDT) technologies. Unlike traditional manual inspection, deep learning enables pixel-level defect detection through the training of semantic segmentation models for various defect features, achieving higher accuracy and consistency. Moreover, lightweight models can be trained for real-time inspection. Several excellent image segmentation architectures have been developed, including the classical U-Net [1], FCN [2], PspNet [3], K-Net [4], DeepLabv3 [5], and Mask2Former [6]. These segmentation networks not only enhance the precision of building pathology diagnosis but also maintain stable performance under complex environmental conditions. Research has shown that techniques such as drone-based dynamic response monitoring, thermal imaging, and deep convolutional neural networks (DCNNs) have significantly improved the accuracy and efficiency of building inspection. Wang, et al. [7] verified that UAV monitoring accuracy can reach up to 2 cm. Zhong, et al. [8] achieved automated diagnosis of façade detachment with an accuracy exceeding 90%; Perez, et al. [9] developed a CNN-based defect detection model using VGG-16 and ResNet-50 with CAM for object localization, supporting real-time detection via mobile devices and UAVs. Dorafshan, et al. [10] demonstrated that DCNNs outperform traditional methods in crack detection accuracy. Additionally, Kung, et al. [11], dos Santos, et al. [12] developed CNN- and Faster R-CNN-based models for façade and roof defect detection, respectively. Liu, et al. [13] proposed a UAV photogrammetry-based damage identification framework using supervoxel segmentation and random forest algorithms, achieving 90% damage identification accuracy. Goessens, et al. [14] validated the feasibility of UAV technology through real building tests, providing a practical reference for subsequent research. El Masri and Rakha [15] reviewed six NDT technologies and their potential applications in building envelope diagnostics. Mayer, et al. [16] used a pretrained Swin-T Transformer model to detect roof thermal bridges, achieving a recall rate of over 50%. Akbar, et al. [17] proposed a UAV-based structural health monitoring system combining SURF and RANSAC, demonstrating robustness to UAV pose displacement and effective displacement detection in real-world structures. Shin, et al. [18] summarized the limitations and improvement directions for UAV–AI hybrid inspection of residential buildings. In the field of infrastructure, Liu and Chou [19] developed an embedded deep learning model for bridge inspection. Li, et al. [20] achieved automatic defect identification in photovoltaic systems. Qiu and Lau [21] integrated YOLO into UAV-based real-time pavement crack detection. Yang, et al. [22] enhanced wind turbine blade damage detection using Otsu threshold segmentation. Ellenberg, et al. [23], Kulkarni, et al. [24] proposed infrared-based methods for detecting pavement voids and bridge deck deterioration, respectively. Tomita and Chew [25] reviewed infrared thermography applications in building delamination detection, evaluating approximately 200 studies and analyzing key factors affecting detection accuracy, providing benchmarks for standardized testing. However, despite the effectiveness of single-modality techniques in specific scenarios, they exhibit notable limitations in complex building environments. Visible-light imagery can clearly capture crack edges but is easily affected by illumination changes, shadows, and reflections, making it difficult to reveal hidden defects such as internal leakage. In contrast, thermal infrared imagery can highlight abnormal temperature regions and is thus suitable for detecting water infiltration and insulation defects, but it suffers from low spatial resolution and environmental sensitivity. Consequently, single-modality approaches cannot comprehensively identify both surface and internal defects, limiting the accuracy and applicability of façade defect detection. Table 1 compares UAV-based building inspection studies. Existing research mainly focuses on individual defect types, achieving 50% to 90% detection accuracy. This study, however, uses RGB-based semantic segmentation for crack detection (92.3% recall) and thermal imaging for leakage detection (86.44% recall) to address two common façade pathologies. Combined with 3D modeling, this approach enables high-precision detection and spatial localization of multiple defect types.

In addition, the influence of UAV aerial photography parameters on inspection efficiency and data quality has increasingly attracted research attention. Tan, et al. [26] proposed a method integrating unmanned aerial vehicles (UAVs) with Building Information Modelling (BIM) to achieve automated surface inspection of buildings. This approach addresses the challenge of maintaining both completeness and high quality of data acquisition while minimizing flight path length, a key consideration given the limited endurance of UAVs. The coverage path planning problem was solved using a genetic algorithm (GA), with inspection areas extracted from the BIM model of the target building. In a subsequent study, Liu, et al. [27] further developed a UAV inspection path planning method that integrates 3D reconstruction with BIM. The proposed workflow includes rough flight for environmental data collection, inspection waypoint calculation, and path optimization, providing a technical foundation for automated building inspection. Similarly, Bolourian and Hammad [28] proposed a LiDAR-equipped UAV path planning method for bridge inspection. Their approach considered the potential locations of defects and employed a genetic algorithm to achieve collision-free trajectories, minimal occlusion, maximum coverage, and shortest flight duration through comprehensive optimization. Ivić, et al. [29] developed a multi-UAV trajectory planning algorithm based on the Heat Equation–Driven Area Coverage (HEDAC) method for 3D visual inspection of complex structures. This method demonstrated significant advantages in reducing operation time, enhancing safety, and improving cost-effectiveness. Nap, et al. [30] combined terrestrial laser scanning (TLS) with unmanned aircraft systems (UAS) for point cloud-based monitoring of large buildings, successfully identifying façade deformations. Schischmanow, et al. [31] proposed a seamless real-time 3D thermal-mapping workflow that integrated visual-inertial navigation with thermal infrared cameras, demonstrating progress toward automated BIM generation. Zheng, et al. [32] in research on façade visual inspection, emphasized that UAV flight paths must ensure adequate building coverage, minimal omission and overlap, and safe distances between the UAV and structures, while optimizing efficiency under these constraints. However, existing studies have paid limited attention to optimizing aerial strategies in high-density urban environments. Significant differences exist among high-rise and multi-story buildings in terms of shooting distance, flight time, data volume, and mapping accuracy. How to design differentiated aerial photography strategies that balance efficiency and precision according to building height remains insufficiently explored. In particular, dense and aging residential areas—characterized by severe occlusion and narrow spaces—still lack targeted solutions for effective UAV inspection.

After automated defect detection, a new challenge has emerged: the detected pathological defect information is stored across a large volume of images, making it difficult to manage and analyze effectively. To address this, several researchers have explored integrating defect information with three-dimensional (3D) models, enabling more efficient management and visualization of large-scale inspection data—now a growing research trend. Chen, et al. [33] proposed a novel approach combining Building Information Modelling (BIM) and UAV-captured aerial imagery for automatic detection and reconstruction of concrete defects. Their method aligns aerial images with the BIM model using a bundle adjustment algorithm, allowing access to building-related semantic material information. This integration reduces false positives caused by irrelevant background objects and significantly enhances defect detection accuracy. Yang, et al. [34] proposed a surface defect-extended BIM generation method combining UAV imagery with deep learning, projecting defects onto BIM models through transfer learning and texture mapping. Tan, et al. [35] developed a method for mapping façade defect data from UAV imagery onto a BIM model. The process involves preprocessing UAV-acquired façade images to extract useful information and introducing a simplified coordinate transformation method to convert real-world defect locations into BIM coordinates. A deep learning-based instance segmentation model was employed to detect and extract defect features from the images. Finally, the identified defects were modelled as new BIM objects with detailed attributes and mapped to corresponding BIM components. Similarly, Pantoja-Rosero, et al. [36] proposed an end-to-end automated workflow for building damage assessment by generating a Level of Detail 3 (LOD3) digital twin enriched with defect information. This method integrates multi-view stereo (MVS), structure-from-motion (SfM), and machine learning models to automatically generate geometric representations of buildings, segment damage regions, and characterize defects. Unlike traditional workflows, this process requires no manual intervention, produces lightweight models, and can be widely applied to various asset types. However, current approaches largely rely on specialized BIM software and lack lightweight, programmable 3D mapping tools accessible to designers and analysts. Particularly during the building renovation and redesign phase, there is still no mature solution for rapidly integrating defect detection results into parametric design platforms such as Rhino Grasshopper. Achieving linked workflows between defect statistics, façade analysis, and renovation planning remains a challenge, which in turn limits the practical application efficiency of inspection results in real-world architectural design workflows.

In summary, although extensive research has been conducted in digital reconstruction of building information and pathological defect recognition and diagnosis, current studies still face several specific technical challenges in façade inspection for existing residential areas in high-density urban environments, including the following: (1) Immature visible–infrared bimodal collaboration methods. Most existing research relies on a single image modality and fails to fully exploit the complementary advantages of visible-light imagery (high spatial resolution) and thermal infrared imagery (temperature sensitivity). In particular, under humid southern climates, methods for jointly identifying two typical façade defects—cracks and water leakage—remain underdeveloped. (2) Insufficient research on differentiated aerial photography strategies in high-density urban contexts. Few studies have systematically examined the trade-offs between data acquisition efficiency and mapping accuracy at different building heights and shooting distances, making it difficult to provide operational guidance for UAV inspections in dense, aging residential areas. (3) Lack of lightweight 3D mapping tools aligned with design workflows. Current BIM-based integration methods primarily depend on specialized software and have not achieved deep interoperability with parametric design platforms such as Grasshopper, thereby limiting the efficiency and flexibility of applying inspection results during the architectural renewal and design phase. Building upon research on existing building regeneration in Shenzhen and previous field investigations, this study proposes a deep learning–based building defect detection and visualization method that integrates visible–infrared data fusion with Grasshopper-based parametric modelling. Through the systematic integration of multimodal imagery collaboration, differentiated UAV strategies, and parametric 3D mapping, this research provides a methodological reference for enhancing the quality and sustainability of existing residential environments in high-density urban settings.

## 2. Materials and Methods

**Figure 1:**
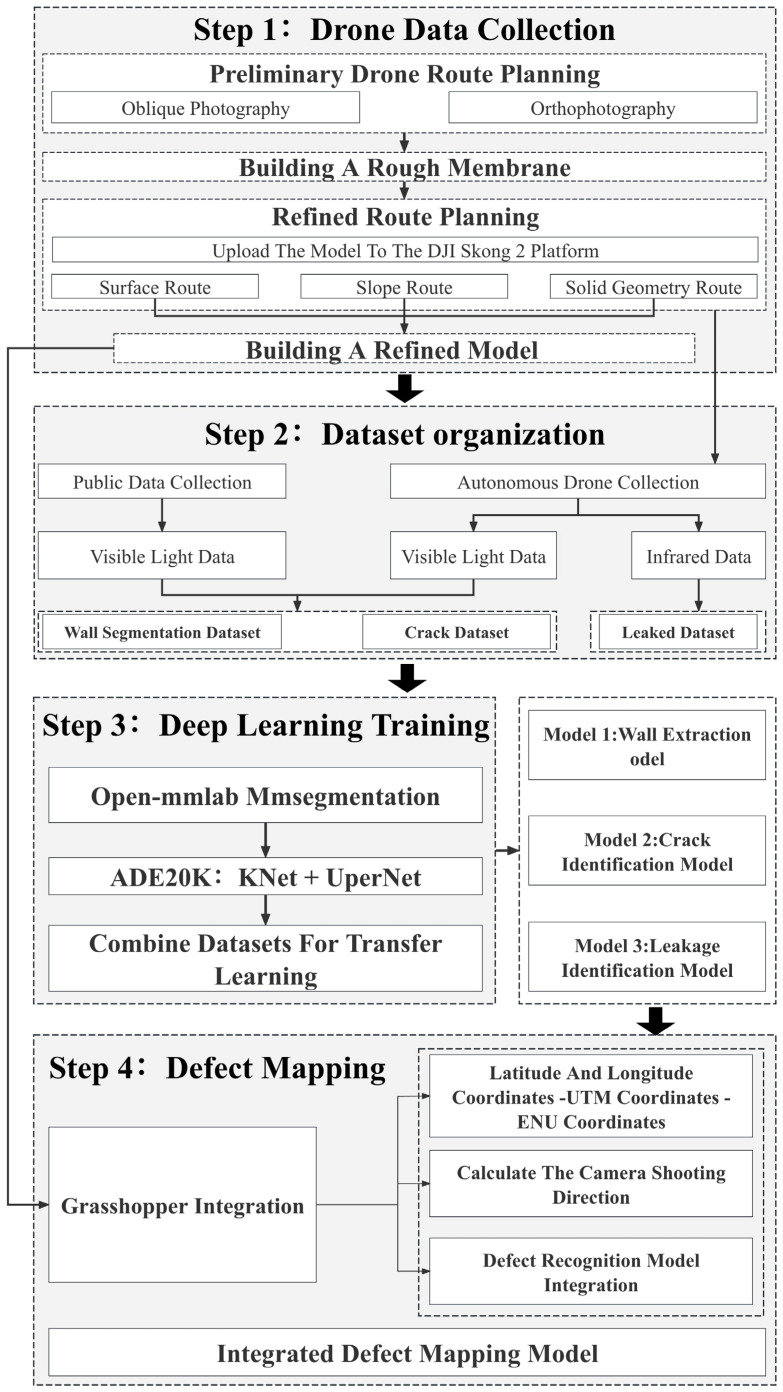
Flowchart of the research methodology.

In this study, UAV aerial photography and deep learning techniques were employed for data acquisition, defect identification, and mapping of façade pathologies in existing residential buildings. A DJI M3T UAV (DJI Innovations Technology Co., Ltd., Shenzhen, China) was used to perform oblique photography of building façade, producing preliminary imagery. Refined flight routes were further planned using the Sikong II platform to capture both visible-light images and infrared thermal images. Subsequently, three specialized deep learning models were trained based on SegFormer, KNet, and UPerNet algorithms: (1) a wall-extraction model, (2) a crack-detection model for visible-light images, and (3) a leakage-detection model for thermal infrared images. Finally, the defect recognition results were spatially mapped with the geoinformation of the images to generate a three-dimensional distribution model of façade defects in residential buildings (Figure 1).

### 2.1. Building Defect Data Collection for Existing Residential Communities in Shenzhen

**Figure 2:**
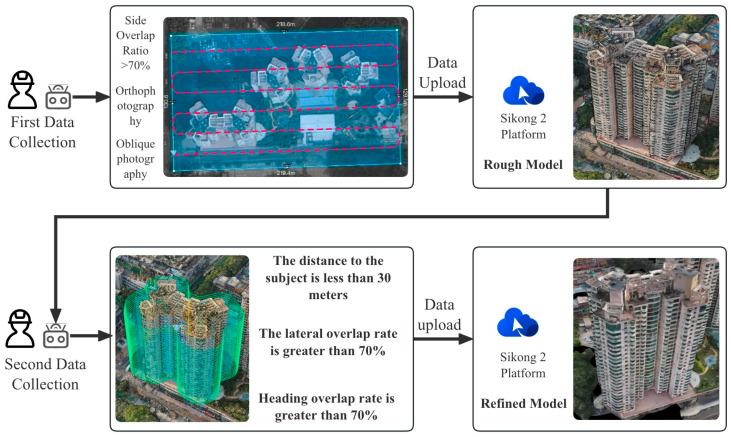
The workflow of data acquisition.

This study focuses on existing residential buildings in Shenzhen constructed between 1988 and 2010, with field investigations conducted to capture façade defects. First, a DJI M3T UAV was employed to perform oblique photography of the target buildings, and preliminary site models were generated using DJI Terra. Subsequently, refined flight routes were planned via the DJI SkyCity platform. In the map interface, inclined or geometric flight paths were rapidly designed by clicking or dragging anchor points. The inclined route automatically adhered to the target façade for stepped or parallel grid layouts, whereas the geometric route encircled individual buildings in spiral or layered grid patterns to ensure full coverage of three-dimensional structures. Flight safety was assessed and optimized in real time through three-dimensional simulation views and elevation profiles. Once finalized, flight routes were automatically exported as KMZ files and synchronized with the UAV controller, enabling an efficient closed-loop process from planning to execution. Using this approach, high-resolution visible-light and thermal infrared datasets were collected, providing a solid foundation for subsequent refined modelling. Thermal infrared data acquisition was scheduled during early morning or late afternoon, when temperature differences between materials are most pronounced, thereby enhancing the contrast of thermal anomalies. Radiometric calibration and temperature correction were applied to minimize environmental interference. Finally, image registration techniques were employed to achieve precise alignment between visible-light and thermal infrared imagery (Figure 2).

### 2.2. Automated Defect Detection Based on Deep Learning

The annotation process involved manually labeling defect regions for the leakage detection task. Temperature anomaly regions indicating moisture intrusion were identified in thermal infrared images and manually annotated using the Labelme tool (https://labelme.io/). For crack detection, a publicly available annotated dataset was utilized, which already contained pixel-level semantic segmentation labels for crack boundaries in visible-light images. These annotated datasets provided precise ground truth labels for training the deep learning models, ensuring reliable supervision during the learning process.

**Figure 3:**
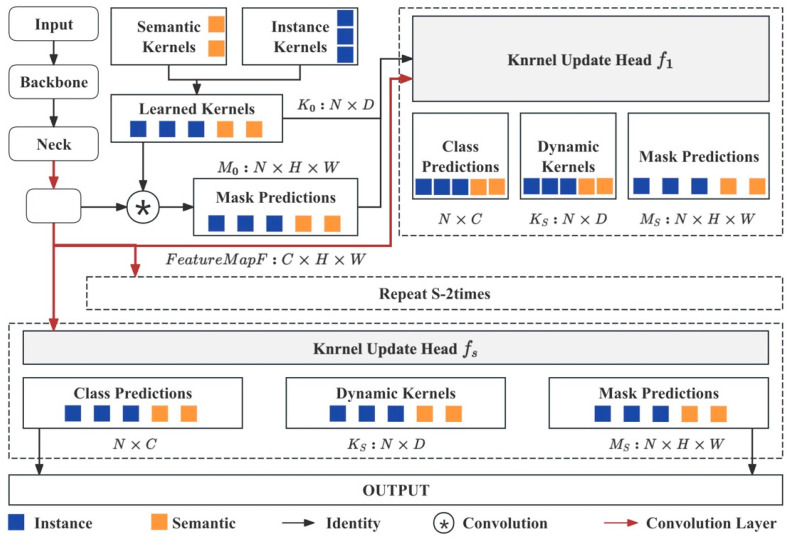
Schematic of the K-Net Algorithm.

Based on the acquired and annotated image data, three specialized deep learning models were trained for automated defect recognition. A wall extraction model was developed using the SegFormer architecture, initialized with a pretrained model on the ADE20K dataset and fine-tuned with the WHU wall dataset (900 images), enabling accurate segmentation and localization of wall regions in building façade. For crack detection, a model combining the KNet and UPerNet architectures was employed, also initialized with ADE20K-pretrained weights and further trained on a publicly available crack image dataset (Figure 3), thereby achieving reliable identification of crack patterns in visible-light façade images. For leakage detection, the same network architecture and pretraining strategy were adopted, but the model was trained with a customized dataset constructed in this study from thermal infrared images.

In the practical detection workflow, the collected visible-light and thermal infrared data were first subjected to wall extraction, after which a sliding-window block detection strategy was applied to the extracted wall regions. Specifically, high-resolution façade images were divided into smaller patches consistent with the resolution of the training datasets, allowing crack and leakage recognition to be performed at multiple scales and ensuring robust detection accuracy. All three models followed the same deep learning architecture and training pipeline, thereby establishing a complete recognition chain from wall extraction to defect identification, which significantly improved the efficiency of façade defect detection in existing buildings. All experiments were conducted on a workstation equipped with an NVIDIA GeForce RTX 4090 GPU ((24 GB VRAM), Santa Clara, CA, USA) and an Intel Core i9-13900K CPU (Santa Clara, CA, USA), with 32 GB of system memory (RAM). The operating system was Windows 11. The deep learning framework used was PyTorch 2.1.0 with CUDA 12.1 support, running on Python 3.10.12.

### 2.3. Three-Dimensional Spatial Mapping of Defects

#### 2.3.1. Spatial Mapping of Visible-Light Data

**Figure 4:**
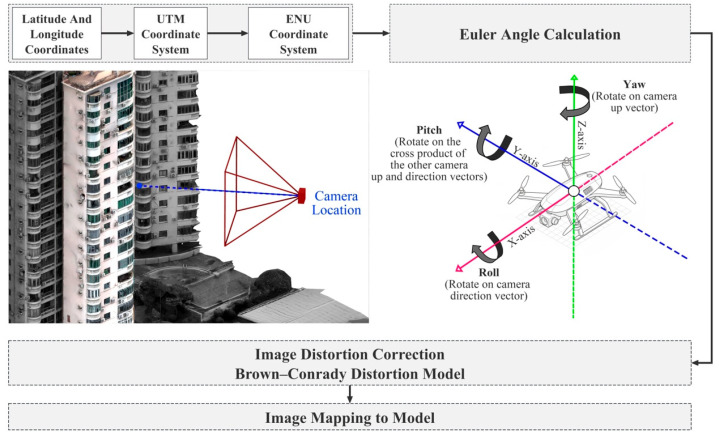
Schematic of Defect Mapping.

**Figure 5:**
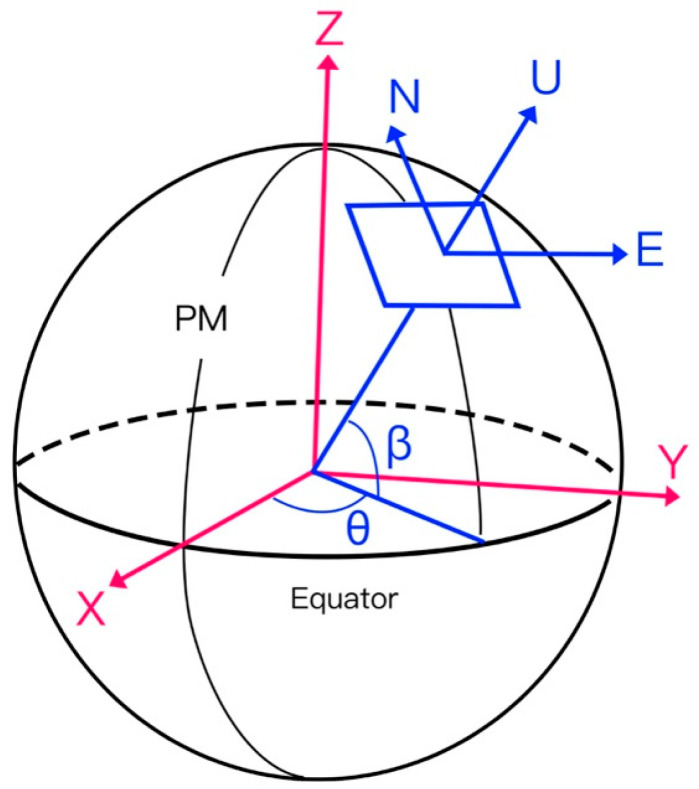
Schematic of the WGS84 Earth Model, ENU Coordinate System, and Their Transformation Relationships (PM denotes the Prime Meridian; β and θ represent WGS84 longitude and latitude; ECEF corresponds to X, Y, Z axes; ENU corresponds to East, North, and Up).

After defect recognition, the results were spatially mapped with the geoinformation of the images. The implementation of this method relies on several key steps, including the parsing of the 3D reality-based reconstruction report, unification of coordinate systems, camera pose estimation, lens distortion correction, and ray generation. First, in terms of coordinate system processing, the WGS84 ellipsoid was adopted as the reference model, where the geographic coordinates (φ,λ) were projected into the UTM coordinate system to obtain $\left(x_{UTM},y_{UTM}\right)$ [37]. Subsequently, reference points extracted from the Structure-from-Motion (SfM) reconstruction report were used to establish a local East-North-Up (ENU) coordinate system, from which the position vector of the camera in the local coordinate system was derived [38,39] (Figure 4 and Figure 5).

(1) $$C_{\mathrm{ENU}}=(X,Y,Z{)}^{T}$$

This approach avoids the nonlinear complexity of geodetic calculations, ensuring consistency in subsequent 3D ray generation. Camera orientation is determined by Euler angles (ω,φ,κ) extracted from the SfM report, from which a rotation matrix R is constructed to describe the transformation between camera and world coordinate systems. The principal viewing direction vector is directly derived from these angles:

(2) $$d=\left[\begin{array}{c} \sin\varphi \\ -\cos\varphi \cdot \sin\omega \\ \cos\varphi \cdot \cos\omega \end{array}\right]$$

To ensure mapping accuracy, the Brown-Conrady model was applied for lens distortion correction [40,41]. This model accounts for both radial distortion (coefficients k_1_, k_2_, k_3_) and tangential distortion (coefficients p_1_, p_2_), with all parameters derived from the camera calibration file. Corrected image coordinates are then converted to normalized camera coordinates by incorporating focal lengths and principal point offsets, enabling the transformation from pixel space to the camera coordinate system (Appendix A).

**Figure 6:**
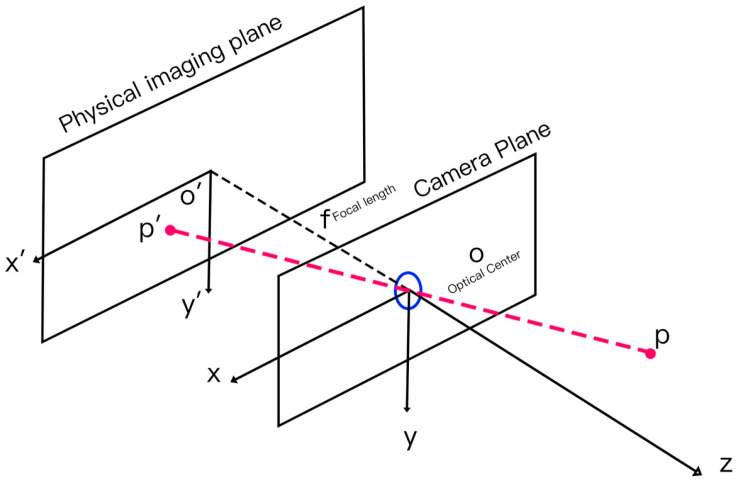
Schematic of the Pinhole Imaging Model.

In this study, the camera is simplified to a pinhole imaging model [42] (Figure 6). For a real-world spatial point Pw(Xw, Yw, Zw), its corresponding coordinates in the camera coordinate system are Pw′(Xw′, Yw′, Zw′), its image plane coordinates are P(x,y), and its pixel coordinates are p(u,v). Under this model, the local ray direction vector is defined as

(3) $$d_{cam}=(x^{\prime },y^{\prime },1{)}^{T}$$

By further applying the camera pose transformation matrix, the ray direction in the world coordinate system can be obtained as

(4) $$d_{world}=R\cdot d_{cam}$$

Thus, defects detected in two-dimensional images can be mapped into three-dimensional space, represented as rays originating from the camera position $C_{ENU}\;$ and oriented along $d_{world}$. The entire implementation was carried out on the Grasshopper platform. By importing the target images and corresponding metadata, the system automatically performed coordinate transformation, pose estimation, distortion correction, and ray generation. The final outputs include the position of the camera in 3D space, the geometric representation of the camera model, and the set of rays corresponding to the annotated defect regions. These results provide the geometric foundation for subsequent spatial analysis and defect modelling.

#### 2.3.2. Three-Dimensional Mapping of Thermal Infrared Images

**Figure 7:**
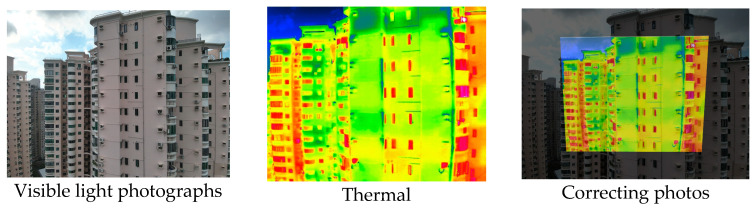
Infrared Thermal Imaging Distortion Correction.

For the 3D mapping of thermal infrared images, it is first necessary to register the infrared thermal image with the visible-light image. In this study, more than four matching points were manually selected, and a homography matrix was computed [43]. A scale-invariant method then adjusts camera intrinsic parameters to match the actual image resolution (Appendix B): focal lengths and principal point coordinates are scaled proportionally, while distortion coefficients remain unchanged due to their dimensionless nature. The corrected pixel coordinates can then be used for 3D ray generation (Figure 7).

## 3. Results

### 3.1. Case Study

In terms of field investigation and data collection, on-site surveys were conducted in the OCT East Cluster, Shennan Garden, and Huifang Garden in Nanshan District, Shenzhen, as shown in Table 1. These three cases exhibit significant differences in construction period, building height, architectural type, and development density, reflecting the typical characteristics of existing residential areas in Shenzhen’s high-density urban environments (Table 2). The OCT East Cluster represents the typical form of Shenzhen’s early low-rise residential buildings. Shennan Garden reflects the high-density development model of the 1990s. Huifang Garden features a more complex architectural form. Together, the three cases cover a range of building types from low-rise to high-rise and from slab-type to tower-type structures. All are located in high-density urban areas with varying degrees of surrounding obstruction and complex environmental conditions, providing representative experimental sites for validating this study.

### 3.2. Multi-Scale Data Acquisition Strategy

This study adopts a two-stage data acquisition strategy of “coarse modelling–detailed acquisition”. The purpose of this strategy is to establish an overall spatial framework through an initial rapid scan, followed by refined data collection for key façades, thereby improving operational efficiency while ensuring inspection accuracy.

#### 3.2.1. Coarse Data Acquisition and 3D Reconstruction

In the first stage, oblique photography was carried out using a DJI M3T UAV to rapidly obtain multi-angle images for generating a basic 3D model of the study area. The flight path parameters were set as follows: 80% forward overlap, 70% side overlap, and a flight altitude of 30 m above the target rooftop. This configuration ensures complete image coverage while maintaining high flight efficiency.

As shown in Table 3, the coarse data were processed using the standard photogrammetric workflow in DJI Terra software (V5.0.0). The procedure included image import and preprocessing (distortion correction and color balancing), followed by aerial triangulation (AT), where geometric relationships between images were established through feature point matching to generate a sparse point cloud. A dense matching algorithm was then applied to produce a high-density point cloud, which was subsequently used for 3D mesh reconstruction and texture mapping.

Although the coarse model lacks sufficient detail for defect identification, it provides an accurate geometric reference framework and spatial positioning basis for the subsequent detailed data acquisition. This enables the fine flight path planning to be optimized based on real 3D terrain conditions.

#### 3.2.2. Detailed Data Acquisition and Differentiated Strategy

In the second stage, the DJI Skysight 2 platform was used for refined flight path planning to obtain high-resolution visible and thermal infrared images. According to the terrain characteristics, building heights, and safety conditions of different study areas, differentiated flight strategies were adopted. In the OCT case, considering the presence of dense tree occlusions and the relatively low number of building stories, a close-range photogrammetry mode was applied with a camera-to-facade distance of 5 m to ensure flight safety. For Shennan Garden and Huifang Garden, under open terrain conditions, the shooting distance was set to 20–25 m, achieving a balance between data accuracy and flight efficiency while effectively controlling the overall operation time, as shown in Table 4.

By comparing the data acquisition parameters of the three cases, it can be observed that building height and shooting distance have a significant impact on operational efficiency and data volume. For low-rise buildings (7 stories) captured at a close range of 5 m, the unit-area flight time was approximately 0.013 h/m^2^. In contrast, for high-rise buildings (over 30 stories) captured at a medium range of 20–25 m, the unit-area flight time decreased to 0.005–0.006 h/m^2^. This result provides a quantitative basis for differentiated aerial photography strategies in high-density urban environments.

### 3.3. Defect Detection

This study adopts MMSegmentation as the main implementation framework. Developed based on PyTorch, it features a modular design that allows customized semantic segmentation models. For façade inspection, three models were trained, respectively, for window–wall recognition, crack detection, and seepage detection, forming a multi-level defect detection process from global façade to local defects, and from visible to thermal infrared data.

To achieve accurate segmentation of building regions, the SegFormer algorithm was used, with transfer learning from a pretrained model on the ADE20K dataset. SegFormer adopts a hierarchical Transformer encoder that effectively extracts multi-scale features, while its lightweight MLP decoder ensures efficient inference. The model performs well in separating buildings from background elements such as sky, vegetation, and roads, providing accurate Regions of Interest (ROIs) for subsequent defect detection (Table 4).

**Figure 8:**
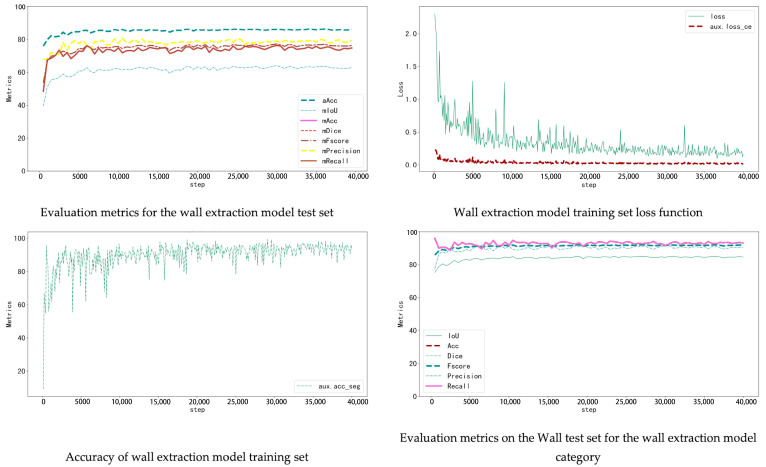
Wall extraction model training indicators.

The window–wall recognition model was trained on the WHU Building Dataset, which includes 900 annotated façade images. The dataset was divided into 720 training and 180 testing samples (8:2 ratio). The model combines K-Net and UPerNet architectures: K-Net learns instance-aware features through dynamic convolution kernels, while UPerNet captures multi-scale context with a pyramid pooling module. Training used ADE20K pretrained weights, running 40,000 iterations with an initial learning rate of 0.0001 and a Poly decay strategy, recording metrics every 500 iterations. The loss function decreased rapidly during the first 5000 iterations and then converged steadily without overfitting. On the test set, the model achieved aAcc 86.11%, mIoU 64.04%, and Dice 77.21%. It effectively distinguishes windows, walls, and other components, providing a solid basis for accurate localization of cracks and seepage (Figure 8).

The crack detection model was trained on a public crack dataset containing 1892 labeled images, divided into 1514 training and 378 testing samples. Given the linear and low-contrast features of cracks, data augmentation such as random cropping, flipping, and color jittering was applied to enhance robustness under different lighting and textures.

**Figure 9:**
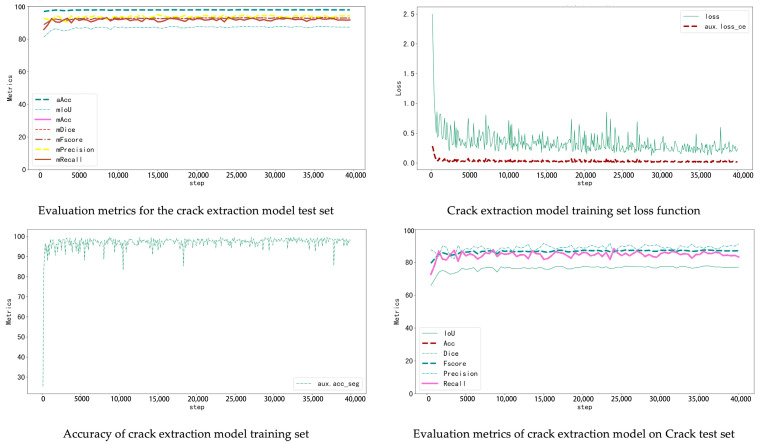
Crack extraction model training indicators.

After 36,000 iterations, evaluation results reached aAcc 98.03%, mIoU 87.86%, and Dice 93.23% (Table 5). The model accurately detects cracks of various widths and orientations and distinguishes them effectively from background textures such as brick joints and decorative lines (Figure 9).

**Figure 10:**
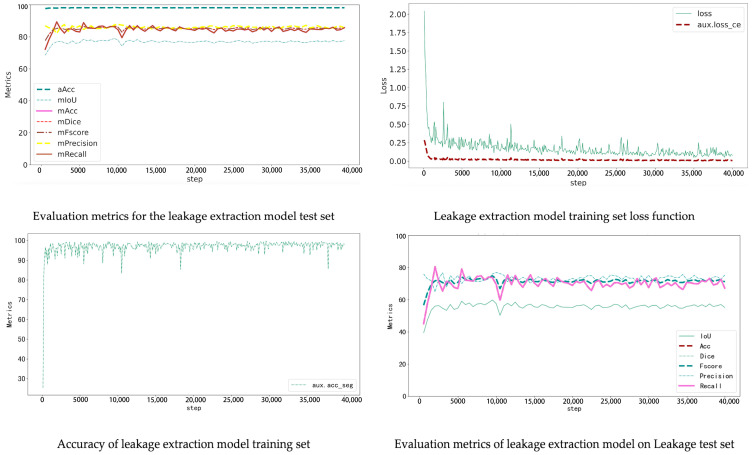
Leakage extraction model training indicators.

The seepage detection model was built using thermal-infrared-assisted annotation. Temperature anomaly regions in thermal images were first labelled automatically and then manually verified based on stains and efflorescence visible in RGB images. This produced a dataset of 340 labelled images. Due to the small dataset size, data augmentation and transfer learning were used to reduce overfitting. The same training parameters as above were applied. The final test results achieved aAcc 98.41 and mIoU 79.05. Although the accuracy is slightly lower than the crack model, incorporating thermal data enables effective detection of early-stage seepage, compensating for the limitations of visible-light imagery (Figure 10).

### 3.4. Defect Mapping

#### 3.4.1. Crack Mapping Process from Visible-Light Images

Based on the trained deep learning models, the collected visible-light images were batch-processed for semantic segmentation to extract both building wall regions and crack defect regions. Table 6 illustrates the processing workflow using four representative images from the Shennan Garden case. First, the building extraction model segmented the main building body, removing background interference. Then, the crack detection model identified crack masks within the wall regions. Finally, the original images were overlaid with the crack masks to generate annotated crack maps. The results show that the model accurately detects cracks of varying widths and orientations, demonstrating strong robustness against wall texture and shadow variations. For example, the vertical crack in DJI001 and the diagonal crack in DJI002 were both successfully extracted, verifying the effectiveness of the deep learning approach under complex background conditions.

#### 3.4.2. Seepage Mapping Process from Thermal Infrared Images

The processing of thermal infrared images is relatively complex, involving distortion correction, visible–infrared registration, and seepage detection. Table 6 illustrates the complete thermal image processing workflow using the same image set. First, the wall regions were extracted from the visible-light images. Then, the corresponding thermal infrared images were loaded and corrected using the Brown–Conrady distortion model to obtain geometrically rectified thermal images. Next, homography matrices were computed by manually selecting feature points to achieve geometric registration between the visible and infrared images. Finally, the seepage detection model was applied to the registered composite images to generate annotated seepage maps. As shown in Table 7, distortion correction significantly improved the geometric accuracy of the thermal images, while the registered fusion images accurately aligned with the wall regions in the visible spectrum. The seepage detection results demonstrate that the model effectively identifies temperature anomaly regions, though some false detections remain and require secondary filtering in combination with visible-light features.

#### 3.4.3. Three-Dimensional Defect Mapping in the Grasshopper Platform

**Figure 11:**
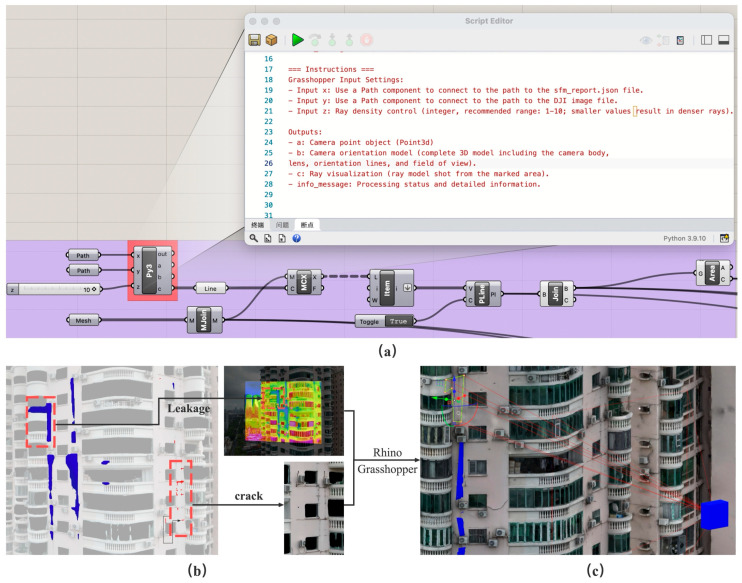
Grasshopper integration: (**a**) use Grasshopper to call the trained model for detection and mapping; (**b**) infrared and visible light collaborative detection; (**c**) defect mapping 3D modelling.

Based on the coordinate transformation and ray projection methods described in Section 2.3, a 3D defect mapping tool was developed within the Grasshopper platform. This tool automatically converts image pixel coordinates into 3D world coordinates by reading camera pose parameters, intrinsic data, and distortion coefficients from the SfM reconstruction report. Table 8 presents the mapping results of four representative cracks from the Shennan Garden case, including crack area, 3D centroid coordinates, and defect 3D models. Through 3D visualization, designers can intuitively understand the spatial distribution and relationships of defects, providing valuable references for repair and renovation planning. Moreover, the parametric nature of the Grasshopper platform allows defect data to be dynamically linked with the architectural geometry model, enabling automated functions such as defect statistics, façade analysis, and data-driven design feedback, significantly enhancing overall design efficiency (Figure 11).

## 4. Discussion

Traditional building façade inspection methods face several inherent limitations. Manual visual inspection is labor-intensive, subjective, and poses safety risks when accessing high-rise buildings. Single-modal automated detection approaches, while improving efficiency, are constrained by their limited sensing capabilities—each modality can only capture specific types of defects and may miss critical information that falls outside its detection range. Furthermore, these methods often lack spatial context and struggle to integrate defect information with building geometry for comprehensive condition assessment. To overcome these shortcomings, researchers have increasingly turned to multimodal data fusion, leveraging the complementary information of different sensors. As early as Ribarić, et al. [44] studies attempted to combine infrared and RGB imagery for façade thermal insulation diagnosis. Lin, et al. [45] integrated thermal imaging and 3D point clouds to develop a high-precision thermal texture mapping method, demonstrating the advantages of cross-modal complementarity. Zhang, et al. [46] designed a hybrid attention-aware fusion network to systematically integrate multimodal building data, confirming the effectiveness of deep-learning-driven fusion. Zhang, et al. [47] reviewed multimodal image fusion methods, and Jabeen, et al. [48] proposed a multimodal deep learning classification framework, further solidifying the theoretical foundation of data fusion. Motayyeb, et al. [49] achieved 87–90% accuracy in thermal leakage mapping by combining thermal infrared and visible-light images, highlighting multimodal potential in energy efficiency assessment. Li, et al. [50] developed MMFNet, a multisensor fusion model achieving high-precision building extraction. Zhou, et al. [51] enhanced structural type recognition through the integration of remote sensing imagery and knowledge graphs, further illustrating the advantages of multimodal fusion in complex scenarios. However, existing studies predominantly focus on algorithm validation, lacking systematic strategies specifically targeting particular defects. This is particularly challenging in high-density urban residential buildings, where the construction of a multi-modal detection system for synergistic use remains an unresolved issue. This study establishes a visible-light–thermal infrared collaborative identification framework for high-density residential buildings. Experimental results indicate that a shooting distance of approximately 20 m from the building achieves an optimal balance, with thermal infrared detecting leakage temperature anomalies and visible light identifying most cracks [52,53]. For visible-light crack detection, illumination conditions and the complexity of façade textures affect detection accuracy. In thermal infrared leakage detection, façade orientation plays a key role: south-facing or sun-exposed façades retain considerable heat in the evening, reducing the temperature differential between leakage hotspots and the background, thus hindering detection. In contrast, shadowed façades create higher temperature contrasts, improving detection sensitivity [54,55]. Additionally, material properties impact thermal infrared performance, as differences in thermal inertia and emissivity between various surface materials cause leakage defects to present inconsistent features in thermal images, increasing the uncertainty in model transfer. During infrared thermal imaging for leakage detection, temperature differences caused by evaporation, capacitance, or conduction are influenced by weather conditions or indoor humidity levels. In subtropical climates with high temperature and humidity, background thermal interference is particularly pronounced, aligning with the observations made in this study in Shenzhen. Therefore, training samples must encompass diverse scenarios (various orientations, materials, and climatic conditions) to improve the model’s adaptability in identifying both types of defects. In comparison with traditional methods, while manual crack gauges and handheld infrared cameras offer high accuracy, their detection range is limited, efficiency is low (requiring several days per building), and the data is discrete [56]. By contrast, the proposed method of combining drone imaging with deep learning offers significant advantages in terms of detection range, efficiency, and data integrity [57,58]. In terms of detection range, drones can cover all areas of the building’s external façade, including high-rise sections, corners, and areas typically inaccessible by traditional methods, achieving full coverage inspections. Regarding efficiency, data collection for a single high-rise building can be completed within 2 h, and the automated processing of defect identification and 3D mapping further shortens the overall inspection cycle. As for data integrity, this method generates continuous 3D defect distribution models [59] (Table 9). However, this method does not yet match the quantitative accuracy of traditional crack gauges. For scenarios where precise crack width measurements are required to assess structural safety, manual verification or close-range supplementary imaging is still necessary. Therefore, a combined approach—using drone imaging for wide-area initial screening followed by traditional crack gauges for detailed measurements of critical areas—may represent a more practical and reasonable technical pathway at this stage.

Experimental results show that different building conditions have significant effects on UAV image acquisition, thermal infrared performance, and 3D mapping accuracy. Building height creates a typical trade-off between detection accuracy and acquisition efficiency. For mid-rise buildings (6–7 stories), when the shooting distance is about 5 m, the acquisition time per building is approximately 1 h, the data volume remains manageable, and a high point cloud density and mapping accuracy can be maintained [60]. In contrast, for high-rise buildings (20–30 stories), if close-range shooting (around 5 m) is still used to capture façade details, the acquisition time extends to 3–4 h, the data volume increases significantly, and the post-processing workload rises sharply. Conversely, when the shooting distance increases to 25–35 m, although the acquisition time can be reduced to about 1.5–2 h, issues such as sparser point clouds, lower thermal image resolution, and increased lens distortion emerge, leading to a decline in overall mapping accuracy [61,62]. Further analysis indicates that a shooting distance of approximately 20 m achieves a better balance for comprehensive detection of two types of façade defects: cracks and water seepage. At this distance, thermal infrared imagery effectively captures temperature anomalies caused by wall seepage, while visible light imagery can still identify most crack defects. This study identifies a technical pathway for high-rise building façade inspection that achieves an optimal balance between efficiency and accuracy. It substantially reduces the time required for data acquisition and processing (Table 10), while maintaining reliable recognition of major defects. Building upon these findings, a comprehensive data acquisition strategy is proposed for façade inspection of high-rise buildings. For low-rise buildings (≤7 stories), a close-range shooting distance of 5–10 m is recommended to obtain high-quality visible light imagery and clear thermal images. For mid-rise buildings (8–18 stories), a shooting distance of 15–20 m is recommended to balance façade coverage and detection accuracy. For high-rise buildings (≥19 stories), a medium-range distance of 20–25 m is recommended as the primary acquisition strategy, which significantly improves acquisition efficiency while ensuring that both sensors can effectively identify major defects. When the shooting distance exceeds 30 m, the detection reliability for cracks and low-temperature-differential leakages deteriorates noticeably due to sensor resolution limitations. In terms of image acquisition timing, periods of direct sunlight and substantial ground heat radiation should be avoided. Experimental observations indicate that early morning or late afternoon is preferable, as these periods strengthen thermal contrast in infrared imagery. Particularly when façades exhibit pronounced orientation differences, multi-temporal sampling is encouraged to enrich the diversity and representativeness of the training dataset. For regions with high temperature and humidity, the timing of image capture should align with local climatic conditions to minimize background thermal interference. At the model training and application stages, incorporating thermal imagery from façades with diverse orientations, materials, and climatic environments can significantly improve the generalization capability and robustness of the inspection model.

In the aspect of multi-modal registration and 3D mapping, this study employed a visible light–thermal infrared registration algorithm, and integrated a Grasshopper-based mapping tool to project and visualize defect detection results onto three-dimensional building models. Although this method achieves stable fusion in most cases, limitations persist due to differences in resolution, baseline distance, and shooting angles between visible and thermal infrared sensors [63,64]. The main sources of mapping error can be summarized as follows. First, when the imaging distance increases, each thermal infrared pixel covers a larger physical area, thereby amplifying leakage localization errors. Conversely, excessively close-range imaging yields denser point cloud data but drastically increases data processing workload, reducing efficiency. Second, common protruding structures on building façades—such as balconies and outdoor air-conditioning units—can decrease the reconstruction accuracy of 3D façade models, making local mapping errors unavoidable. These elements cause occlusion and shadow effects, which compromise the completeness of point cloud generation and result in positional deviations of defect projections on the 3D model. Furthermore, geometric transformation errors accumulated during the registration process between visible and thermal infrared images also propagate into the final 3D mapping results. Because the two sensors differ significantly in field of view (FOV), resolution, and lens distortion characteristics, registration deviations may still occur in geometrically complex regions such as building edges and corners, even after distortion correction. To enhance registration stability, it is recommended to incorporate camera distortion parameters and radiometric emissivity corrections of the thermal imager during the registration process. Additionally, in the 3D modelling phase, increasing image overlap, adopting oblique viewing angles, and deploying ground control points (GCPs) can further improve the overall accuracy and absolute georeferencing capability of the 3D model.

While this study provides methodological and technical foundations for multi-modal façade inspection, we acknowledge that achieving truly systematic application across diverse urban contexts depends on factors beyond the scope of this research. These include the development of standardized operational protocols, regional cost–benefit assessments, integration with existing municipal maintenance management systems, and validation across different building typologies, construction periods, and climatic conditions.

The proposed method is specifically designed for large-scale building inspection scenarios, with particular suitability for large-scale residential area surveys, municipal-level building safety inspections, and preliminary diagnostics for urban renewal projects. This framework is positioned as a comprehensive first-stage screening tool rather than a replacement for detailed structural safety assessments. This study recommends a two-tiered strategy combining UAV-based wide-area screening with targeted manual detailed inspection: the first stage employs the proposed method to conduct rapid comprehensive screening across extensive building façades, identifying defect locations and preliminary severity levels; the second stage involves traditional manual inspection and quantitative measurement of critical defect areas identified in the initial screening. This approach achieves an optimal balance between inspection coverage and precision in large-scale applications, meeting the practical needs of municipal authorities for systematic, periodic safety monitoring of extensive building stocks.

## 5. Conclusions

In response to the urgent demand for façade defect detection in high-density urban residential buildings, this study proposes a systematic inspection framework integrating drone-based visible and thermal infrared imaging, deep learning algorithms, and parametric 3D visualization. Through empirical studies conducted in three typical residential communities in Shenzhen (constructed between the 1980s and 2000s), the proposed method was validated for its effectiveness in the collaborative detection of cracks and leakage defects. The main conclusions are as follows:

First, multi-modal fusion enables differentiated and synergistic recognition of cracks and leakage defects. Visible-light imagery effectively captures the geometric characteristics of cracks, while thermal infrared imagery detects temperature anomalies associated with leakage. However, thermal infrared detection is sensitive to façade orientation, material properties, and climatic conditions. Therefore, incorporating diversified training samples is crucial to enhance model adaptability and robustness across varying environments.

Second, there exists a trade-off between mapping efficiency and accuracy depending on building height and imaging distance. For low-rise buildings, close-range imaging ensures high mapping precision, whereas for high-rise structures, medium-range imaging at approximately 20–25 m achieves the optimal balance between detection efficiency, accuracy, and dual-defect recognition capability.

Third, the developed Grasshopper-based integrated mapping tool enables effective 3D visualization and parametric analysis. This tool can be seamlessly embedded into design workflows for rapid application. The main sources of mapping error stem from variations in imaging distance, façade occlusions by architectural components, and sensor discrepancies. A “coarse screening–fine measurement” strategy—first conducting wide-area preliminary inspection using drone imagery, followed by targeted manual measurement at critical locations—represents a practical and efficient technical pathway for current engineering applications.

Despite these contributions, several limitations remain: (1) The empirical validation was limited to three communities in Shenzhen, and the model’s generalizability to different geographic regions, construction periods, and structural types has yet to be verified. (2) The current framework focuses on two defect types—cracks and leakage—without addressing other common issues such as delamination or spalling. (3) Mapping precision still requires improvement in geometrically complex regions such as building edges and corners.

Future research should focus on several key directions. First, integrating emerging sensing technologies such as radar and hyperspectral imaging will expand multimodal data sources, improve the characterization of complex defect features, and further enhance mapping accuracy. Second, the detection framework should be extended to cover additional defect types, including hollowing and spalling, through the adoption of multi-task learning architectures that enable comprehensive and scalable façade health assessment. Third, refining error evaluation methodologies and optimizing registration algorithms are essential to address precision bottlenecks in geometrically complex regions and to enhance the overall robustness and applicability of the proposed system in real-world engineering contexts. Moreover, with the continuous advancement of building maintenance technologies, future studies could broaden the inspection scope to include lower façade zones and high-rise viewpoints, thereby enriching the spatiotemporal dimensions of façade defect diagnostics. Overall, this research establishes a technical foundation for intelligent façade defect detection in existing residential buildings within high-density urban environments. The proposed system framework and optimization strategies provide a scientific basis for urban planners and building managers to develop sustainable maintenance systems, contributing substantially to the improvement of urban building safety, resilience, and management efficiency. The proposed framework provides a practical foundation for developing automated building maintenance systems and promoting their integration into future smart city infrastructures.

## Figures and Tables

**Table 1:** Comparison of existing inspection methods and research gap analysis.

Study	Detection Method	Sensors	Defect Types	Accuracy Metrics	3D Model
Zhong et al. [8]	UAV IRT	Thermal	Facade debonding	F1: 90%	No
Dorafshan et al. [10]	Edge detection	RGB	Concrete cracks	F1: 89%	No
Kulkarni et al. [24]	Object detection	Thermal	Road subsurface voids	Recall: 51%	No
Zoe et al. [16]	Semantic segmentation	RGB	Concrete surface cracks	Recall: 50%	No
This Study	Semantic segmentation	RGB + Thermal	Cracks + leakage	Cracks Recall: 92.3% Leakage recall: 86.44%	Yes

**Table 2:** Information on Research Sites.

Site	Construction Time	Number of Layers	Building Type	Floor Area Ratio	Number of Houses	Number of Households
OCT	1987	7	Slab Building	3.5	27	1152
Shennan Garden	1995	34	Slab Building	6.7	4	642
Huifang Garden	1994	33	Tower	3.8	4	984

**Table 3:** Initial data acquisition.

Site		Coarse Film-Route Planning	Survey Area	Flight Time	Number of Photos	Building A Rough Model
**OCT**	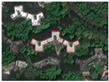	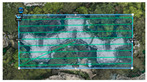	2504.91 m^2^	5 min	130	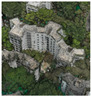
**Shennan Garden**	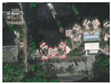	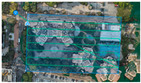	14,089.91 m^2^	9 min	240	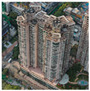
**Huifang Garden**	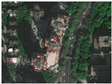	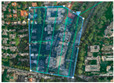	50,517.54 m^2^	15 min	460	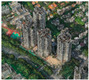

**Table 4:** Refined data acquisition.

	OCT	Shennan Garden	Huifang Garden
Refined route planning	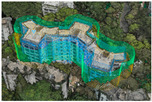	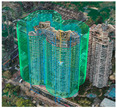	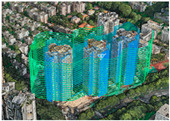
Survey area	3471.6 m^2^	18,368.73 m^2^	29,100.71 m^2^
Flight time	45 min	1.2 h	1.6 h
Distance from the subject	5 m	22 m	25 m
Number of visible light photos	1034	1400	1811
Infrared quantity	1034	1400	1811
Number of batteries (pieces)	1	2	3
Average discharge ratio per battery	90%	80%	75%
Battery capacity per unit (mAh)	5000	5000	5000

**Table 5:** Training results of each model.

	aAcc	mIou	mAcc	mFscore	mPrecision	mRecall	Step
Wall extraction model	86.11	64.04	76.33	77.21	78.9	76.33	30,000
Crack extraction model	98.03	87.86	92.31	93.23	94.21	92.31	36,000
Leakage extraction model	98.41	79.05	86.44	86.98	87.54	86.44	9500

**Table 6:** Crack Mapping Results.

OCT	DJI0001	DJI0002	DJI0255	DJI0049
Visible light image	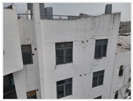	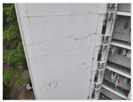	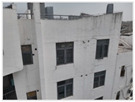	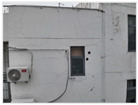
Semantic segmentation results	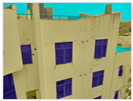	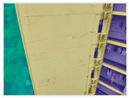	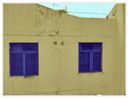	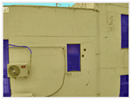
Infrared image	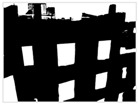	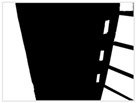	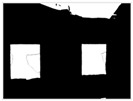	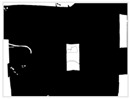
Crack mask extraction	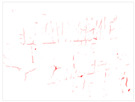	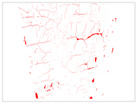	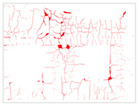	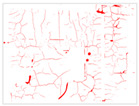
Crack extraction	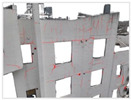	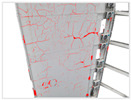	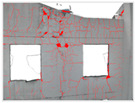	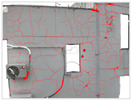

**Table 7:** Leakage Mapping Results.

Shennan Garden	DJI0001	DJI0002
Visible light image	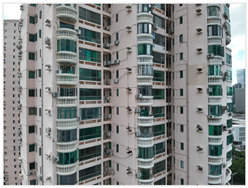	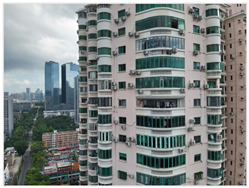
Infrared image	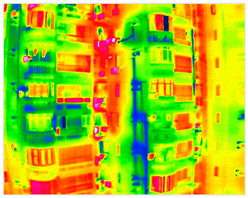	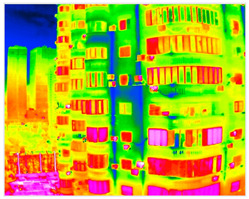
Infrared distortion correction	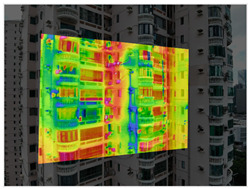	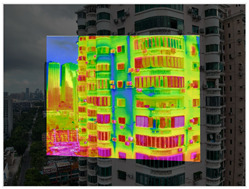
Detection image	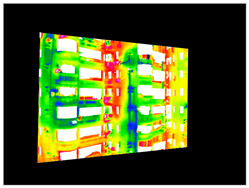	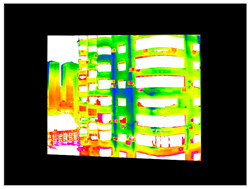
Test results	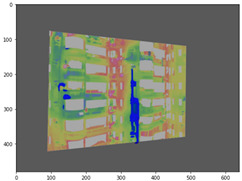	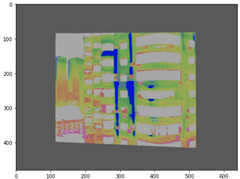

**Table 8:** Defect Modelling Results.

	Leakage01	Leakage02	Leakage03	Leakage04
Area	0.470811	0.156714	0.245465	0.916632
Pathology Coordinate Center	14.069919, −28.201071, −4.560413	14.366629, −35.160761, −1.445286	14.134377, −28.100611, 3.877598	8.378084, −29.317375, −3.580256
Defect Modelling	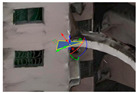	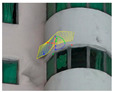	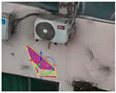	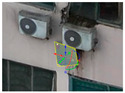

**Table 9:** Compared with traditional methods.

Index	Traditional Crack Meter	UVA + Deep Learning Method
Detection range coverage	About 40–60% (accessible areas only)	≥95% (whole building)
Detection efficiency (single high-rise building)	2–3 Days	2 h
Quantitative measurement accuracy (crack width error)	±0.1 mm	±1 cm
Data integrity (spatial continuity)	★★ ^1^	★★★★★ ^2^
Security	★★ ^1^	★★★★★ ^2^
Degree of human involvement	90%	20%

^1^ Star ratings indicate data integrity, with ★★ representing insufficient integrity; ^2^ Star ratings indicate data integrity, with ★★★★★representing high integrity.

**Table 10:** Data collection efficiency comparison.

Building Type	Shooting Distance	Data Collection Time	Data Scale	Main Impact
Overseas Chinese Town (7th floor)	10 m	~1 h	Suitable	High precision and acceptable efficiency
Shennan Garden (34th floor)	5 m	3–4 h	Too large	High accuracy but extremely low efficiency and high data redundancy
Shennan Garden (34th floor)	15–20 m	~1.5–2 h	Suitable	Improve efficiency, but reduce reconstruction accuracy

## Data Availability

The original contributions presented in this study are included in the article; further inquiries can be directed to the corresponding author.

## Appendix A

Combining a vector with a rotation matrix allows projecting rays from the camera coordinate system into the world coordinate system. To avoid deviations when mapping defects onto the 3D model, lens distortion inherent in UAV cameras must be addressed. This study applied the Brown–Conrady distortion model to correct the image coordinates. For normalized image coordinates (x,y), the radial distortion correction term is expressed as follows:

(A1) $$\Delta r=k_{1}r^{2}+k_{2}r^{4}+k_{3}r^{6}$$

(A2) $$r^{2}=x^{2}+y^{2}$$

$k_{1},k_{2},k_{3}$ denote the radial distortion coefficients. The tangential distortion correction terms are given as

(A3) $$\Delta x=2p_{1}xy+p_{2}\left(r^{2}+2x^{2}\right),\Delta y=p_{1}\left(r^{2}+2y^{2}\right)+2p_{2}xy$$

where $p_{1}$ and $p_{2}$ are the tangential distortion coefficients. The corrected image coordinates can thus be expressed as

(A4) $$\left(x_{c},y_{c}\right)=\left(x+\Delta x,y+\Delta y\right)$$

All of these parameters are derived from the camera intrinsic file. After distortion correction, the coordinates are converted into normalized camera coordinates. Given the pixel coordinates (u,v), principal point coordinates $\left(c_{x},c_{y}\right)$, and focal lengths $f_{x},f_{y}$, the normalized coordinates are expressed as

(A5) $${\;x}^{\prime }=\frac{u-c_{x}}{f_{x}},y^{\prime }=\frac{v-c_{y}}{f_{y}}$$

## Appendix B

Using the homography matrix, the thermal infrared image can be accurately registered to the visible-light image, resulting in a “visible-light + infrared overlay” image. Further, the registered infrared thermal image is subjected to distortion correction. A scale-invariant method was applied to adjust the camera intrinsic parameters. First, the relevant intrinsic parameters, including focal lengths $f_{x},f_{y}$, principal point coordinates $\left(c_{x},c_{y}\right)$, and distortion coefficients $\left(k_{1},k_{2},p_{1},p_{2}\right)$, are extracted, assuming these parameters were obtained at an A×B image resolution. To adapt to the actual resolution of the input image C×D, the scaling factors $scale_{x}$ and $scale_{y}$ are computed as

(A6) $$scale_{x}=\frac{C}{A},scale_{y}=\frac{D}{B}$$

Based on this scaling factor, the new “effective intrinsic parameters” can be computed as follows:

(A7) $$f_{x}^{\prime }=f_{x}\times scale_{x},f_{y}^{\prime }=f_{y}\times scale_{y}$$

(A8) $$c_{x}^{\prime }=c_{x}\times scale_{x},c_{y}^{\prime }=c_{y}\times scale_{y}$$

At the same time, the distortion coefficients $\left(k_{1},k_{2},p_{1},p_{2}\right)$ remain unchanged because they are dimensionless and not affected by image resolution. With these “effective intrinsic parameters,” the image distortion correction is performed. Through this process, the original pixel coordinates undergo a geometric transformation, resulting in valid pixel coordinates that meet the requirements for 3D ray generation.
